# A Case of Immediate Hypersensitivity Reaction to Maltitol

**DOI:** 10.1155/2017/2127167

**Published:** 2017-08-15

**Authors:** Ana Rodríguez Trabado, Carmen Cámara Hijón, José Antonio García-Trujillo, Irene Magriz Trascón, Luis Miguel Fernández Pereira

**Affiliations:** ^1^Allergy Department, Nuestra Señora del Prado Hospital, Talavera de la Reina, Toledo, Spain; ^2^Immunology Department, San Pedro de Alcántara Hospital, Avda. Pablo Naranjo, S/N, 10002 Cáceres, Spain

## Abstract

**Background:**

Maltitol is a sugar alcohol that is frequently used as a noncaloric sweetener, although it is also used as an excipient, a plasticizer in gelatin capsules, and an emollient. It has not been previously described as an agent involved in immediate hypersensitivity reactions.

**Methods:**

We report on an anaphylactoid reaction with pharyngeal occlusion suffered by a 60-year-old man after ingestion of a candy containing maltitol syrup. A prick-to-prick test was performed with the candy and maltitol powder. Other allergens were excluded as causative agents of the adverse reaction, although the patient refused to undergo an oral challenge test with the candy. A basophil activation test (BAT) was performed with maltitol powder, and a dose-response curve was generated. The test was also performed in 3 healthy controls.

**Results:**

Both prick-to-prick tests were negative. The result of the BAT was positive at all the concentrations tested in the patient's blood and negative in all the controls.

**Conclusions:**

The BAT can help to clarify the agents implicated in an adverse reaction and can reduce the risk involved in diagnosis. The BAT can also prove useful in the study of reactions caused by low-molecular-weight antigens, for which routine diagnostic tests are not feasible.

## 1. Background 

**Figure 1:**
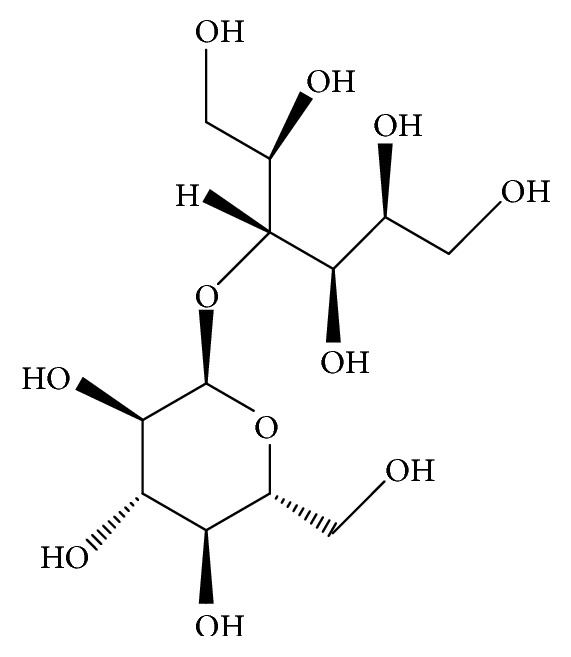
Chemical structure of maltitol (C_12_H_24_O_11_).

Maltitol (4-O-*α*-glucopyranosyl-D-sorbitol) is a sugar alcohol (polyol) that is produced by hydrogenation of maltose obtained from starch (Figure 1). It is frequently used as a noncaloric sweetener because it has half the calories of sucrose (table sugar). It is also used as an excipient in drugs, a plasticizer in gelatin capsules, and an emollient.

Sugar alcohols rarely cause hypersensitivity reactions, although there have been reports of reactions to mannitol [1–4] and erythritol [5]. Maltitol has not been previously described as an agent involved in immediate hypersensitivity reactions.

## 2. Patients and Methods

We report the case of a 60-year-old man with a history of hypothyroidism and cutaneous psoriasis. He reported an acute episode of dyspnea, facial flushing, and pharyngeal occlusion (anaphylaxis grade 2 of Ring and Messmer) immediately after licking a candy (Virginia's Coffee Candies®, Rodríguez S. A. Industries, Reus, Spain). He spat the candy out without swallowing and reported spontaneous improvement after 15 minutes. The candy was composed of maltitol syrup, hydrogenated fatty acids, and coffee. The patient reported that he usually ate the mentioned kind of candies and assessed good tolerance to one of the same candies five hours before the episode and to four units the day before. After the adverse reaction, the patient referred good tolerance to coffee and the other foods taken during the hours preceding the episode.

Prick testing was performed for common inhalants,* Anisakis*, Pru p 3, and a complete battery of foods, including egg, milk, nuts, fruits, legumes, fish, seafood, flour, and spices.

Prick-to-prick testing was first performed with the candy implicated in the adverse reaction.

Prick-to-prick test was later performed with maltitol powder (Sweet Pearl P200, Roquette Laboratories, France).

The general blood analysis included immunoglobulins, 24-hour urinary catecholamines, erythrocyte sedimentation rate, tryptase, anti-thyroid antibodies, and total IgE and specific IgE for* Anisakis* and* Ascaris*. As the patient refused to undergo a controlled oral challenge with the candy, in vitro tests were performed in order to correlate the suspicious components with the clinical picture.

**Figure 2:**
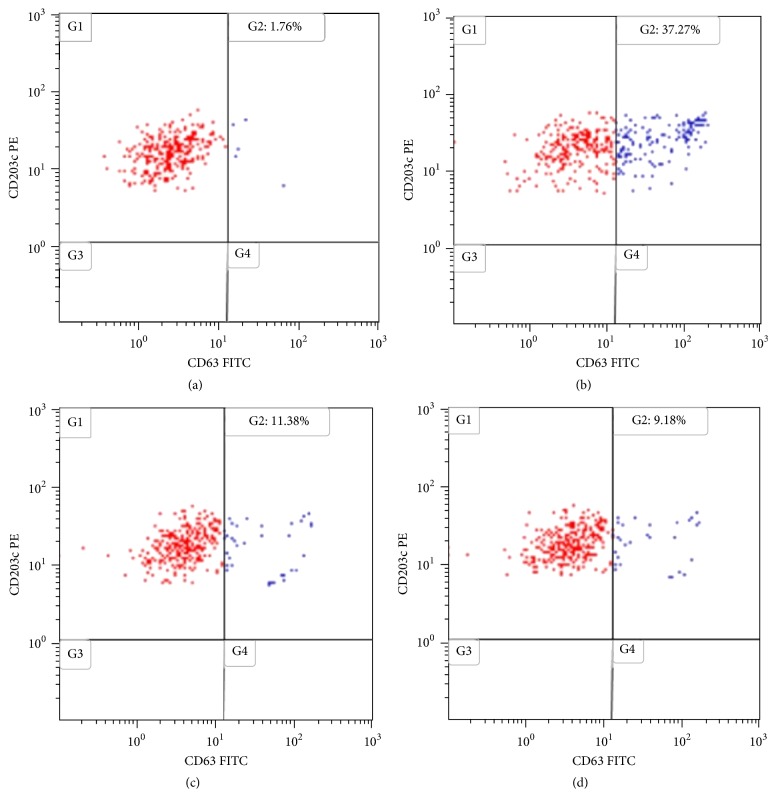
Basophil activation test with maltitol. The G2 quadrant of the dot plots represents the percentage of basophils that expresses CD63 in high intensity (activation of cells). (a) Negative control; (b) positive control; (c) maltitol at 1000 *µ*g/ml; (d) maltitol at 100 *µ*g/m.

The basophil activation test (BAT) was performed with maltitol powder (Sweet Pearl P200) according to a previously reported technique [6, 7]. Briefly, whole blood was drawn, and the analysis was performed within 24 hours. Maltitol was tested in a dose-response curve from 1000 *μ*g/ml to 100 *μ*g/ml. Double staining was carried out with CD203c-PE to detect basophils and CD63-FITC to detect basophil activation (Figure 2). Serum saline was added as negative control (Figure 2(a)), showing the basally activated basophils, before adding any stimulus. The peptide fMLP was used as positive control (Figure 2(b)), to asses an adequate cellular reactivity. The positivity criterion was the same as that applied for other low-molecular-weight substances, such as drugs [6]. Therefore, to consider a result positive, the percentage of basophils that became activated after incubation with maltitol had to be at least double that of the negative control (basophil activation index ≥ 2).

The test was also performed in three healthy individual controls.

## 3. Results

The results of the prick and prick-to-prick tests were negative. Total IgE was 85.3 kU/l and specific IgE was negative for* Anisakis* and* Ascaris*.

The blood analysis revealed no pathological values, except for a slight increase in thyroid peroxidase antibodies (anti-TPO) (109 IU/L, normal values < 35 IU/L).

The result of the BAT was positive at all the concentrations tested (Figure 2 and Table 1). In the background, 1.76% of basophils were CD63 positive (G2 of Figure 2(a)). Maltitol at 1000 ug/ml (Figure 2(c)) induced a basophil activation of 11.38% (activation index 6.46) and maltitol at 100 ug/ml (Figure 2(d)) induced 9.18% activation (activation index 5.21).

In three healthy controls, maltitol did not induce a basophil activation (activation indices 0.55, 1.23, and 1.02) (Table 1).

## 4. Discussion

In this case, the other main components of the candy had been tolerated by the patient after the reaction, and no other food allergies were found. So, maltitol was suspected as the cause of the anaphylactoid reaction.

Other low-molecular-weight sugars have been reported as allergens causing anaphylaxis [1–5]. In general, the capability of low-molecular-weight elements to cause sensitization depends on their binding to proteins to form a hapten-carrier complex. Thus, reactions have been reported between D-glucose and N-terminal amino groups of in vivo proteins, such as serum albumin, hemoglobin, and plasma proteins [8].

Nevertheless, sugars such as mannitol do not have the reactive group necessary for covalent binding to proteins and production of a stable complex [4]. In one case of anaphylaxis due to this sugar, the formation of a Schiff base with the reduced sugar form (D-mannose) and amino groups of proteins has been proposed as a mechanism of sensitization [4]. This base could expose the sugar epitopes, leaving them available as antigenic determinants to induce specific IgE production [4, 9]. The hypersensitivity reaction could then take place by bivalent, or even monovalent, binding to mannitol [10].

## 5. Conclusion

The potential ubiquity of maltitol as well as the absence of feasible routine diagnostic tests to study the hypersensitivity reactions which it could cause means that it is difficult to diagnose as a potential cause of anaphylaxis. The BAT could be a useful and noninvasive technique for the study of cases such as the present one, in which the challenge test involved risk of anaphylaxis and was refused by the patient. Although we were unable to determine the underlying pathogenic mechanism of the reaction, our findings draw attention to the role of maltitol as a causative agent.

## Figures and Tables

**Table 1:** Results of basophil activation and activation index.

	% basophils CD63+	Activation index	Result
Patient			
Negative control	1.76		
Positive control	37.27		
Maltitol 1000 *µ*g/ml	11.38	6.46	Positive
Maltitol 100 *µ*g/ml	9.18	5.21	Positive
Control 1			
Maltitol 1000 *µ*g/ml		0.55	Negative
Maltitol 100 *µ*g/ml		0.42	Negative
Control 2			
Maltitol 1000 *µ*g/ml		1.23	Negative
Maltitol 100 *µ*g/ml		0.87	Negative
Control 3			
Maltitol 1000 *µ*g/ml		1.02	Negative
Maltitol 100 *µ*g/ml		0.92	Negative

## Abbreviations

anti-TPO: peroxidase antibodies
BAT: Basophil activation test
FITC: Fluorescein isothiocyanate
PE: Phycoerythrin.
